# Synthesis, Structure, and Function of Heparan Sulfate Glycopolymers to Investigate Glycosaminoglycan–Protein Interactions

**DOI:** 10.1021/acs.accounts.5c00844

**Published:** 2026-02-16

**Authors:** Kartikey Singh, April Sweet Tapayan, Israel Vlodavsky, Hien M. Nguyen

**Affiliations:** † Department of Chemistry, 2954 Wayne State University, Detroit, Michigan 48202, United States; ‡ Technion Integrated Cancer Center, Rappaport Faculty of Medicine,Technion—Israel Institute of Technology, Haifa 1096, Israel

## Abstract

Heparan sulfate (HS), a highly sulfated glycosaminoglycan, varies in its disaccharide units, chain length, and sulfation patterns. HS structural diversity and its localization at cell surfaces and in the extracellular matrix enable HS interaction with a breadth of HS-binding proteins (HSBPs), HS thus being a co-receptor for other proteins and initiating various biological responses. Several designed and studied HS mimetics modulate HSBP activity implicated in various diseases. A key HSBP is heparanase (HPSE), which can cleave HS into smaller fragments, facilitating release of angiogenic growth factors, activating biological signals that may contribute to pathological conditions (promoting tumor development and metastasis), and enabling autoreactive immune cells to target insulin-producing β-cells. Thus, HPSE serves as a crucial target for disease therapy strategies. Several saccharide-based HS mimetics, designed as HSPE inhibitors, have advanced to clinical trials, but these sugar molecules were discontinued or suspended due to adverse effects from off-target HSBP interactions. Glycopolymers engineered to incorporate functionalized glycan residues into their polymeric backbones are a promising approach to retain endogenous HS’ native biological activity, thereby enhancing therapeutic efficacy. Stereoselective formation of α-1,2-*cis*-glycosidic linkages that connect the glucosamine unit to the uronic acid disaccharide core is challenging during development of HS mimetics as HPSE inhibitors. Computational modeling and a stereoselective catalytic glycosylation method were used to design and synthesize glycopolymer-based HS mimetics with repeating units of the glucosamine–glucuronic acid disaccharide core and a controlled degree of polymerization and incorporate glycan residues with precisely tailored sulfation patterns. This strategy ensures targeted biological activity and maintains structural specificity toward its intended HSBP. Glycopolymers were synthesized using ring-opening metathesis polymerization with the third-generation Grubbs catalyst, enabling precise control over both the degree of polymerization and molecular weight by tuning the catalyst loading. The most potent glycopolymer displayed superior potency and selectivity compared to previously reported monovalent and polymeric HPSE inhibitors and demonstrated remarkable antimetastatic activity in models of mammary carcinoma and myeloma cancer. It also protected pancreatic β-cells and human islets from HPSE-induced damage, suggesting a possible diabetes therapeutic agent. Prioritizing multivalency and precise structural control in polymeric HS mimetics facilitates targeted interactions with specific HSBPs and enhances their potential for precision therapeutic applications.

## Graphical abstract

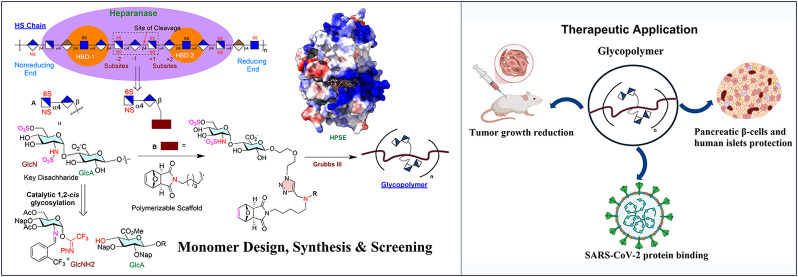

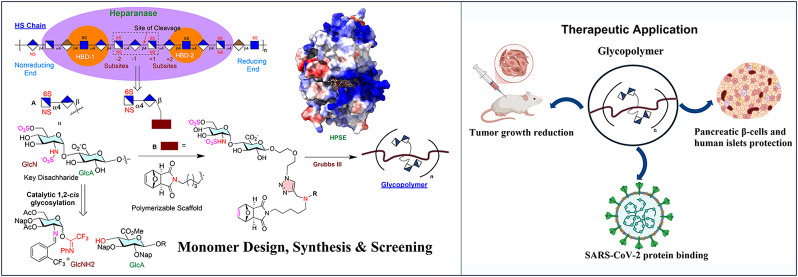

## Key References

-

Sletten, E. T. ; Loka, R. S. ; Yu, F. ; Nguyen, H. M. Glycosidase Inhibition by Multivalent Presentation of Heparan Sulfate Saccharides on Bottlebrush Polymers. Biomacromolecules 2017, 18 (10), 3387–3399.28846389 10.1021/acs.biomac.7b01049PMC6044434.^1^ *This work describes the design and synthesis of a series of well-defined monoantennary and biantennary pendant heparan sulfate saccharide glycopolymers, displaying the effect of length and multivalency on heparanase inhibition.*
-

Loka, R. S. ; Sletten, E. T. ; Barash, U. ; Vlodavsky, I. ; Nguyen, H. M. Specific Inhibition of Heparanase by a Glycopolymer with Well-Defined Sulfation Pattern Prevents Breast Cancer Metastasis in Mice. ACS Appl. Mater. Interfaces 2019, 11 (1), 244–254.30543095 10.1021/acsami.8b17625PMC6512314.^2^ *This work described the structure-based synthesis and identification of the most potent biantennary HS-mimetic glycopolymer heparanase inhibitor, its selectivity over off-target HS-binding proteins, and the high effectiveness of this multivalent inhibitor in inhibiting breast cancer metastasis in vivo.*
-

Sletten, E. T. ; Tu, Y.-T. ; Schlegel, H. B. ; Nguyen, H. M. Are Brønsted Acids the True Promoter of Metal Triflate Catalyzed Glycosylations? A Mechanistic Probe into 1,2-*cis*-Aminoglycoside Formation by Nickel Triflate. ACS Catal. 2019, 9, 2110–2123.31819822 10.1021/acscatal.8b04444PMC6900934.^3^ *This work describes the in situ generation of triflic acid from nickel triflate, which serves as the active catalyst species in glycosylation. Experimental evidence from control reactions and* *^19^F NMR spectroscopy has been obtained to confirm and quantify the triflic acid released from nickel triflate, which is of paramount importance for achieving stereoselective 1,2-cis-2-aminoglycosidic bond formation via a transient anomeric triflate.*
-

Singh, K. ; Tapayan, A. S. ; Sletten, E. T. ; Loka, R. S. ; Barash, U. ; Vlodavsky, I. ; Nguyen, H. M. Heparanase-Inhibiting Polymeric Heparan Sulfate Mimetic Attenuates Myeloma Tumor Growth and Bone Metastasis. ACS Appl. Bio Mater. 2025.10.1021/acsabm.5c0077140663002.^4^ *This study explored HS-mimicking glycopolymers as HPSE inhibitors for multiple myeloma. One glycopolymer inhibited HPSE, reduced myeloma cell viability, and prevented ECM degradation. In vivo, it significantly inhibited tumor growth, surpassing SST0001, and showed efficacy against metastatic myeloma.*

## 1 Introduction

Heparan sulfate (HS) is a highly sulfated glycosaminoglycan composed of a linear polysaccharide that is typically covalently linked to a core protein, forming heparan sulfate proteoglycans (HSPGs). HSPGs are ubiquitously expressed on the cell surfaces and within the extracellular matrix (ECM) of mammalian tissues.^5^ HS is composed of repeating disaccharide units of d-glucosamine (GlcN), which can be either *N*-acetyl-d-glucosamine (GlcNAc) or d-glucosamine-*N*-sulfate (GlcNS), and uronic acid, which can be either d-glucuronic acid (GlcA) or, to a lesser extent, l-iduronic acid (IdoA). The GlcN unit is linked to the uronic acid (GlcA/IdoA) via an α(1→4) glycosidic bond, while the uronic acid unit is connected to another GlcN unit through a β(1→4) linkage (Figure 1A).^5–7^ HS polysaccharides exhibit subtle variations in stereochemistry, length, and sulfation patterns, resulting in significant structural diversity.^8^ This variability, along with HS’s localization on the cell surface and within the ECM, enables it to interact with and regulate the activity of numerous HS-binding proteins (HSBPs). These interactions, in turn, influence a broad range of biological processes, including cell signaling, immune responses, and pathogen invasion. As a result, several HS mimetics have been designed and synthesized to modulate the activity of HSBPs implicated in various diseases.

**Figure 1:**
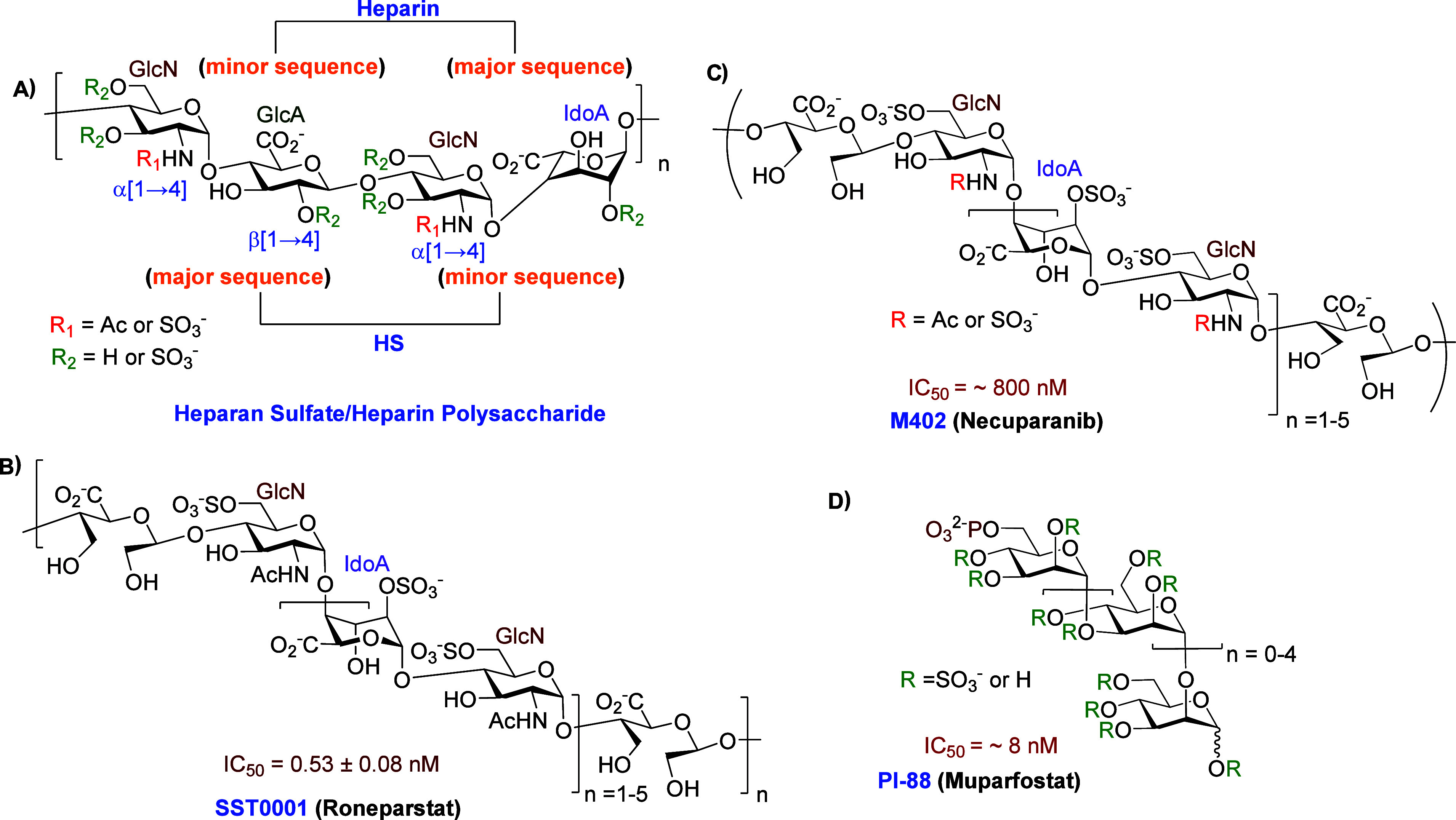
(A) HS, heparin, and HS mimetic clinical candidates: (B) SST0001, (C) M402, and (D) PI-88.

A significant challenge in elucidating the structure–activity relationships of HS and advancing HS-based therapeutic strategies lies in its inherent chemical complexity and heterogeneous sulfation pattern. Although alternatives such as low-molecular-weight HS mimetics have been developed, they face significant limitations in replicating the crucial biological properties of endogenous HS polysaccharides, particularly in maintaining target specificity and avoiding cross-bioactivity.^9,10^ HSBPs recognize and bind to specific glycol-epitopes within the HS chains, a process highly dependent on the spatial arrangement of sulfate groups and the multivalent nature of the polysaccharide backbone, which enhances affinity and selectivity through cooperative binding interactions.^11^

While some studies have focused on modifying natural polysaccharides or semisynthesizing HS mimetics, these approaches lack the structural precision of chemical synthesis, particularly in controlling sulfation patterns and structural variations. To address these limitations, recent efforts have developed well-functionalized glycan residues on a polymeric backbone—known as glycopolymers—as mimetics of native HS polysaccharides.^12^ These synthetic multivalent carbohydrate-functionalized ligands from well-designed respective monomers are facile to assemble and have been shown to retain key biological properties of the natural HS polysaccharides. Nevertheless, this approach remains challenging due to the difficulties associated with the glucosamine–uronic acid disaccharide core via the stereoselective α-1,2-*cis*-2-amino glycosidic linkages.^13,14^ To overcome this hurdle, we developed a method for the synthesis of 1,2-*cis*-2-aminoglycosides via nickel-catalyzed α-selective glycosylation with C(2)-*N*-substituted benzylidene glycosamine donors. This approach enabled us to design and construct a library of glycopolymer-based HS mimetics, with the repeating units of the glucosamine–glucuronic acid disaccharide core, targeting HSBPs associated with various diseases.^15^

Heparanase (HPSE), an *endo*-β-d-glucuronidase, is a key HSBP that regulates the biological functions of HS. HPSE is the sole enzyme that cleaves the β(1→4) glycosidic bond between GlcA and GlcN of HS, resulting in the release of biologically active HS fragments. These fragments act as coreceptors for various chemokines and growth factors, facilitating their release and initiating downstream signaling patways.^16^ Overexpression of HPSE has been associated with numerous pathological conditions, including cancer, inflammation, neurodegeneration, and diabetes. Due to HPSE’s central role in HS structural remodeling and signal modulation, HS-based therapeutics targeting HPSE represent a promising approach for regulating HSBP activity in various pathologies driven by HS–HSBP interactions.^17^ This account provides an overview of the structure-based design and synthesis of structurally diverse glycopolymers as HS mimetics using our stereoselective catalytic α-1,2-*cis*-2-amino glycosylation method to construct the disaccharide core. The subsequent evaluation of their binding and inhibitory activities against HPSE and different HSBPs has yielded insights into the influence of sulfation patterns, degree of polymerization, and other scaffolds on their biological properties.

## 2 HS Mimetic Clinical Candidates: Requiring New Directions

HS is structurally related to heparin, a highly sulfated GAG that has been used as an anticoagulant since 1935.^18^ However, heparin differs from HS by having a higher proportion (∼70%) of GlcN-IdoA disaccharides (Figure 1A). Heparin exerts its anticoagulant effect by binding to antithrombin-III (ATIII), enhancing its ability to inhibit thrombin and factor-Xa, thereby preventing clot formation. Due to its structural similarity to HS, heparin has been identified as a potent HPSE inhibitor. However, its clinical utility is limited by bleeding risks and the potential for heparin-induced thrombocytopenia (HIT).^19^ One HPSE inhibitor candidate, SST0001 (Roneparstat), is a modified heparin synthesized through desulfation and controlled glycol splitting. It is fully *N*-acetylated at GlcNAc and 25% glycol-split at the GlcA units, with a molecular mass of 18–20 kDa (Figure 1B). The minimized presence of IdoA lowers its anticoagulant activity, while the glycol splitting enhances its resistance to HPSE-mediated hydrolysis. SST0001 has completed Phase I clinical trials for multiple myeloma; however, it inadvertently interacts with platelet factor-4 (PF4), triggering an autoimmune response that may cause thrombocytopenia and thrombosis.^20^ Another HS mimetic, M402 (Necuparanib), is a modified heparin derived through controlled depolymerization and functional group modification (Figure 1C).^21,22^ It progressed to Phase II trials as a combination therapy for metastatic pancreatic cancer but was discontinued due to adverse effects and lack of efficacy. PI-88 (Muparfostat), a mixture of sulfated, monophosphorylated mannan oligosaccharides, advanced to Phase III trials for hepatocellular carcinoma (Figure 1D). While it exhibits strong HPSE inhibition, its significant anticoagulant activity and strong binding to other HSBPs have led to side effects such as thrombocytopenia and excessive bleeding, limiting its clinical viability.^23^ These challenges highlight the need for next-generation HS mimetics that can effectively inhibit HPSE while minimizing off-target interactions. One promising approach is to utilize computational modeling to guide the design of precisely sulfated synthetic HS mimetics that modulate HPSE activity without interfering with coagulation pathways. Recent advances in precision chemical synthesis and SAR optimization have facilitated the development of engineered synthetic glycopolymers that function as HS mimetics. These synthetic glycopolymers are composed of monomeric units with well-defined sulfation patterns, which are tuned to enhance target specificity.

## 3 Inspiration for Monomer Design

HPSE plays a key role in HS cleavage, making it crucial to examine the SAR of the HS–HPSE axis when designing HS mimetics. The active form of HPSE is a heterodimer composed of both 8 and 50 kDa subunits. Its domain architecture includes a (β/α)_8_ domain, flanked by a smaller β-sandwich domain, with both subunits playing a role in maintaining the structural integrity of these domains (Figure 2A).^24^

**Figure 2:**
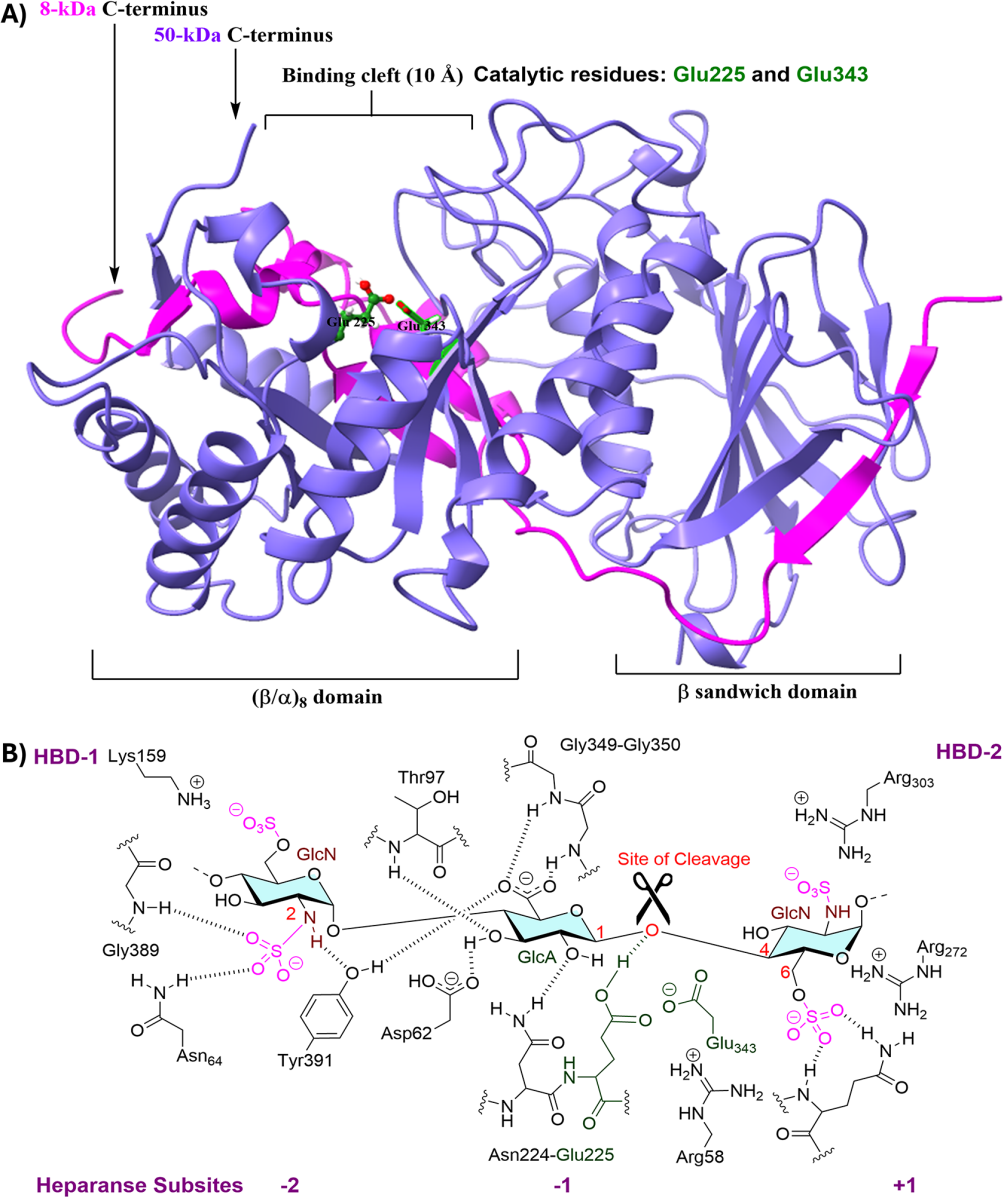
(A) Crystal structure of HPSE (PDB: 5E8M). (B) Representative HS-binding domain with catalytic site.

The catalytic site of HPSE is flanked by two highly positively charged heparan-binding domains (HBD-1 and -2). Structural analyses derived from the cocrystallized structures of HPSE bound to HS ligands reveal that a trisaccharide (GlcNα­(1,4)­GlcAβ­(1,4)­GlcN), which spans −2, −1, and +1 subsites, is the minimum recognition sequence (Figure 2B). The active site of HPSE involves Glu225 (proton donor) and Glu343 (nucleophile), which are key catalytic residues for cleaving the scissile β-(1,4)-linkage between GlcA at the −1 subsite and GlcN at the +1 subsite. 6-*O*-Sulfation at +1 subsite and *N*-sulfation at −2 subsite on the GlcN moieties are essential for recognition.

Drawing from the HPSE–HS recognition pattern, we selected the GlcNS­(6S)­α­(1,4)­GlcA unit of the −2/–1 subsites as the pendant saccharide moiety for our monomer design. Removing the cleavable GlcAβ­(1,4)­GlcN moiety would maintain HPSE recognition but potentially prevent HPSE-mediated hydrolysis of the molecule. Additionally, linking GlcNS­(6S)­α­(1,4)­GlcA to a polymerizable strained bicyclic oxonorbornene ring introduces crucial π-cation interactions, thus enhancing affinity toward HPSE, especially when a triazole moiety is used as the scaffold (Figure 3A). Docking of M1 (Figure 3B) to the *apo* crystal structure of HPSE^24^ demonstrated that the GlcNS­(6S)­α­(1,4)­GlcA disaccharide moiety is positioned into −1/–2 subsite of HPSE (Figure 4A) in a similar fashion to Davies’ cocrystallized HS-HPSE structures (Figure 2B).^24^ Moreover, the *N*-sulfate at −2 GlcNS formed a strong trivalent hydrogen bonding network with Asn64, Gly389, and Tyr391. The 6-*O*-sulfate of the GlcNS ring also formed a hydrogen bond to Arg303 at the −2 subsite. The attached aliphatic scaffold of M1 was located within the binding groove, establishing a network of hydrophobic, π-cation, and hydrogen bonding interactions.^2,22^ Notably, the triazole contributed several of these π-cation and hydrogen bonding interactions with the HPSE active site. Conversely, the strained bicyclic ring of the scaffold typically remained positioned above the binding groove.^1^

**Figure 3:**
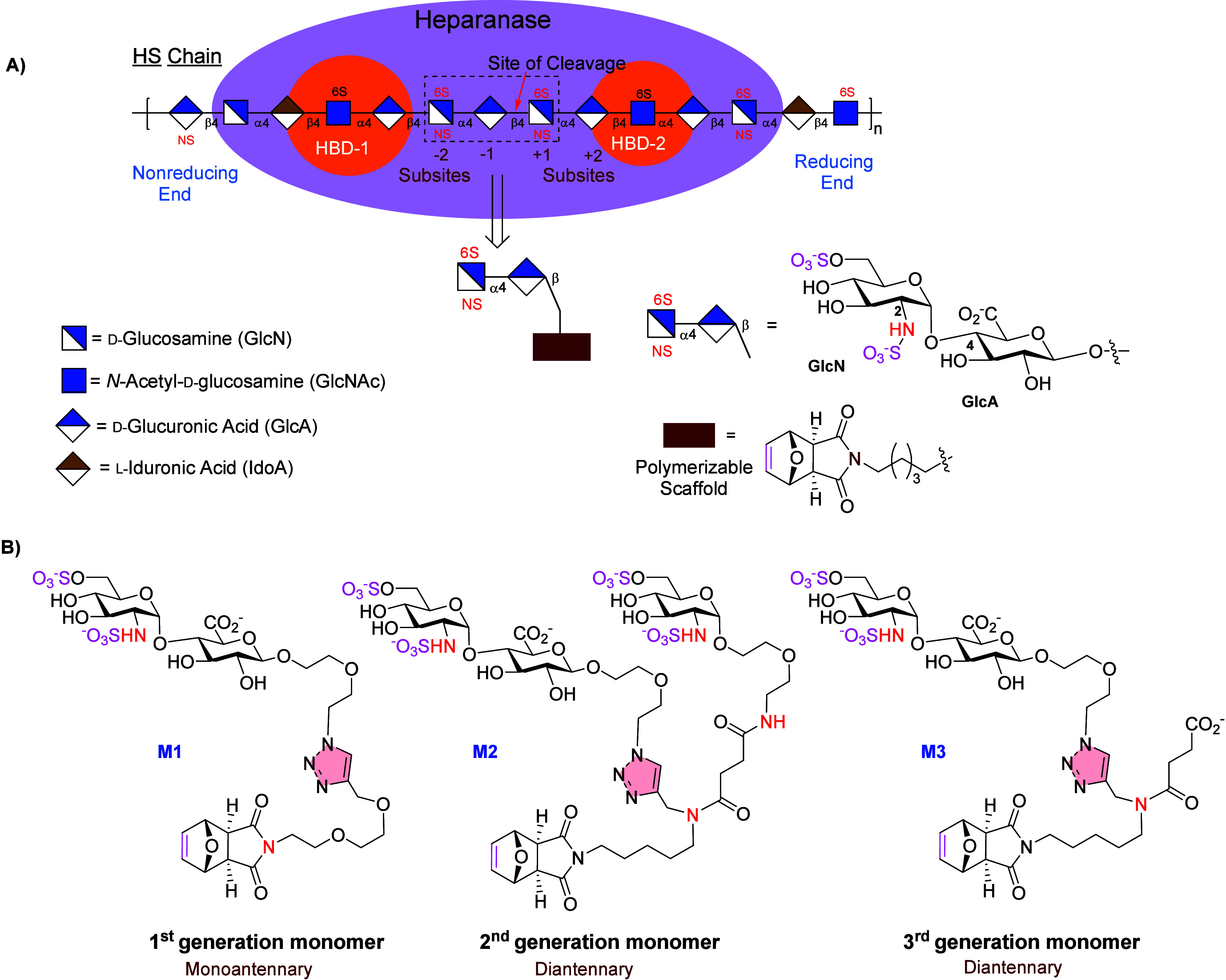
(A) Designing monomers based on a natural HS chain disaccharide glycotope with polymerizable scaffold. (B) Designed monomers.^2,25^

**Figure 4:**
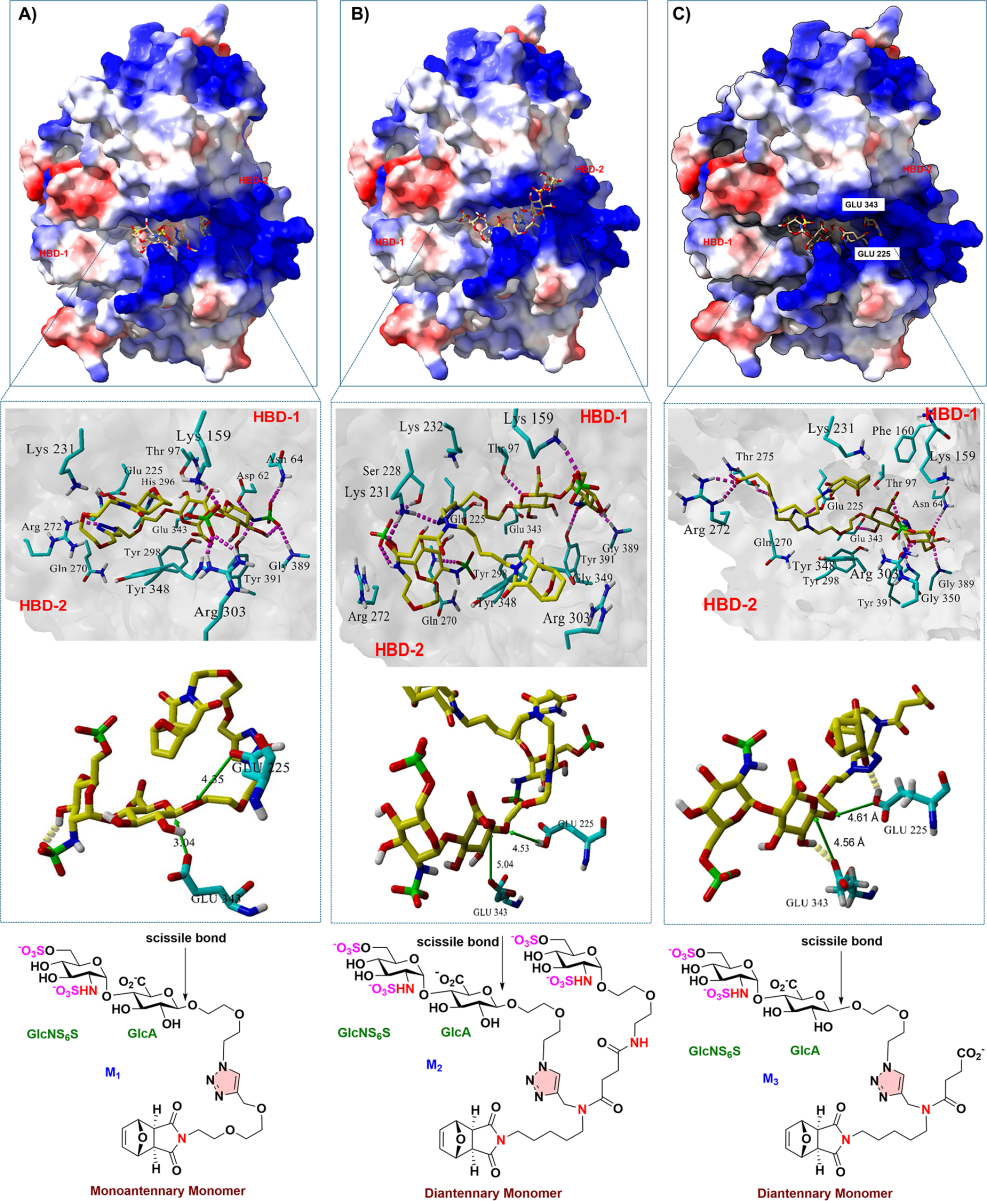
Molecular docking study of the monomeric component of the glycopolymers: (A) M1; (B) M2; and (C) M3 into the crystal structure of the human HPSE enzyme (PDB: 5E8M).^2,25^

We also explored the impact of increasing the local saccharide density on HPSE inhibition by comparing the HPSE binding mode of diantennary monomer M2 with that of monoantennary monomer M1. The additional GlcNS­(6S) in M2 interacts with HBD-2 by forming multiple hydrogen bonds and an ionic salt bridge between the C6-sulfate and Arg272 (Figure 4B). In contrast, M1 exhibits only minimal interactions with HBD-2. During molecular dynamics simulations, the cleavable bond of M2 is found to be positioned farther and skewed away from the catalytic residues, Glu225 and 343, suggesting hydrolysis is unlikely to occur. In contrast, the cleavable bond of M1 adopts an alignment and distance more favorable for nucleophilic attack by Glu 343.^1^

Interestingly, when we removed the GlcNS attachment and replaced the diantennary scaffold of M2 with a simple carboxylate group (M3, Figure 4C), we observed that the CO_2_^–^ group of the GlcA sugar interacts with Arg 272 and Thr 275 in the HBD-2 of HPSE’s active site (Figure 4C). The binding mode is also positioned such that the 6-*O*-SO_3_^–^ of the GlcNS­(6S) sugar unit interacts with Asn64, Gly389, and Tyr391 in HBD-1. The cleavable bond present in the monomer unit of M3 is positioned at an increased distance from Glu225 and Glu343, thereby preventing it from HPSE hydrolysis.^25,26^ These insights allowed us to design and synthesize the first-generation monoantennary (M1),^1^ second-generation (M2) and third-generation (M3) diantennary monomers (Figure 3B). Furthermore, given that M3 exhibited the most favorable interactions with HPSE, coupled with its comparatively straightforward synthetic approach, we proceeded to investigate the impact of varying the sulfation pattern on its GlcN unit by designing a new class of diantennary monomer M4 (Figure 5).^2^

**Figure 5:**
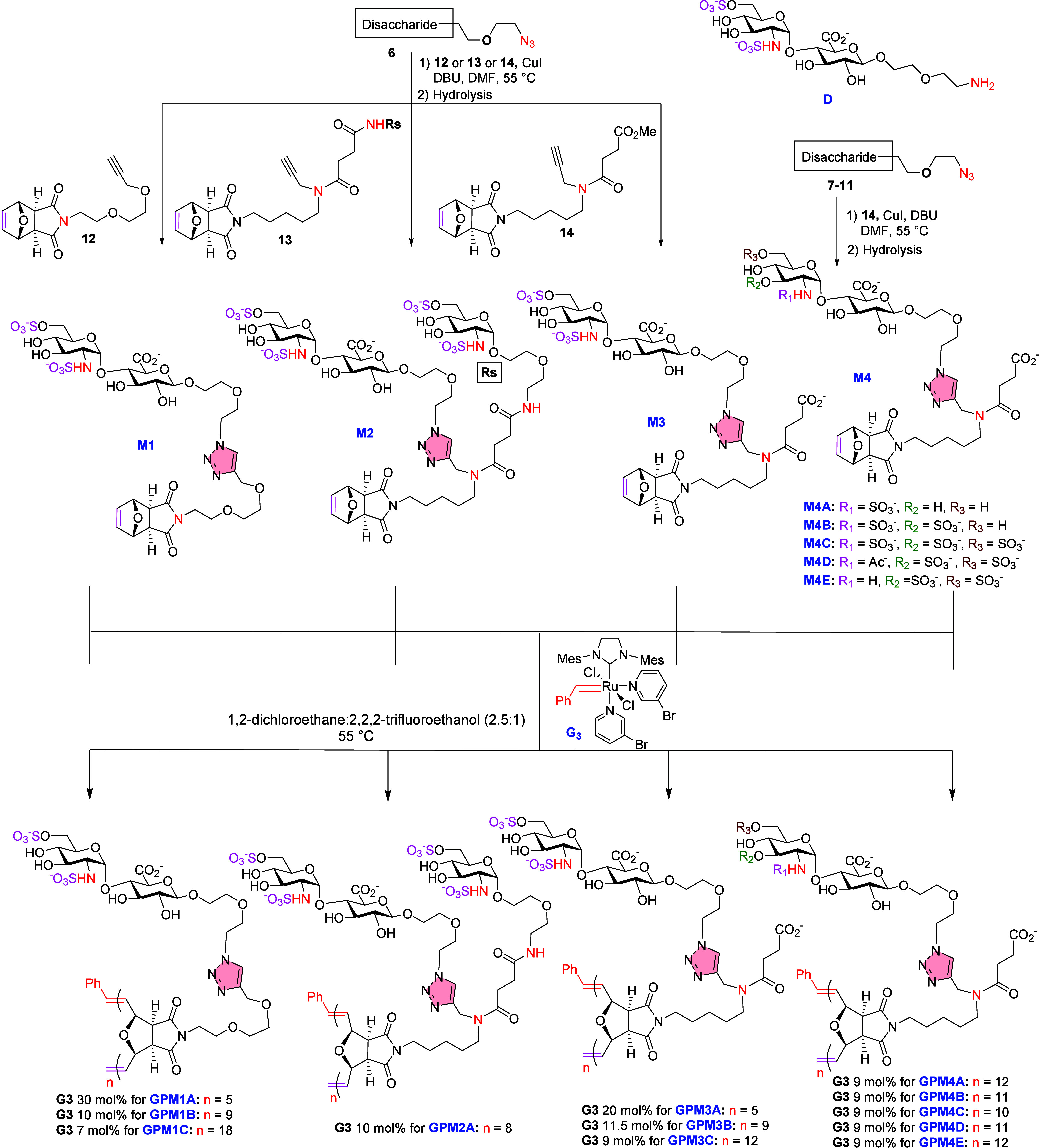
Synthetic route for HS-mimicking glycopolymers from corresponding monomers.

## 4 Synthesis of Designed HS-Mimicking Polymers

A central challenge in the synthesis of HS mimetics is the stereoselective construction of α-1,2-*cis* glycosidic linkages, particularly those involving 2-amino-2-deoxysugars, GlcN (Figure 3A). This is further complicated by the intrinsically low nucleophilicity of the 4-hydroxyl of d-glucuronic acids, GlcA (Figure 3A) acceptors. To address this gap, we developed a catalytic stereoselective glycosylation approach using C(2)-*N*-benzylidene d-glucosamine *N*-phenyl trifluoroacetimidates (Scheme 1) activated by Ni­(OTf)_2_.^15,27^ This method enables the highly α-1,2-*cis* selective synthesis of a variety of bioactive oligosaccharides featuring 1,2-*cis*-2-amino linkages under mild conditions.^28–35^ Mechanistic studies revealed that triflic acid (TfOH), generated in situ from Ni­(OTf)_2_, serves as the activating species.^3^ Variable temperature NMR experiments revealed a glycosyl triflate-like intermediate, and DFT calculations combined with a kinetic analysis support a mechanism in which triflic acid facilitates the formation of this intermediate. This, in turn, allows isomerization from a stable α-anomer to a reactive β-anomer, facilitating S_N_2-like displacement by nucleophiles to produce 1,2-*cis*-2-aminoglycosides (Scheme 1).

**Scheme 1:**
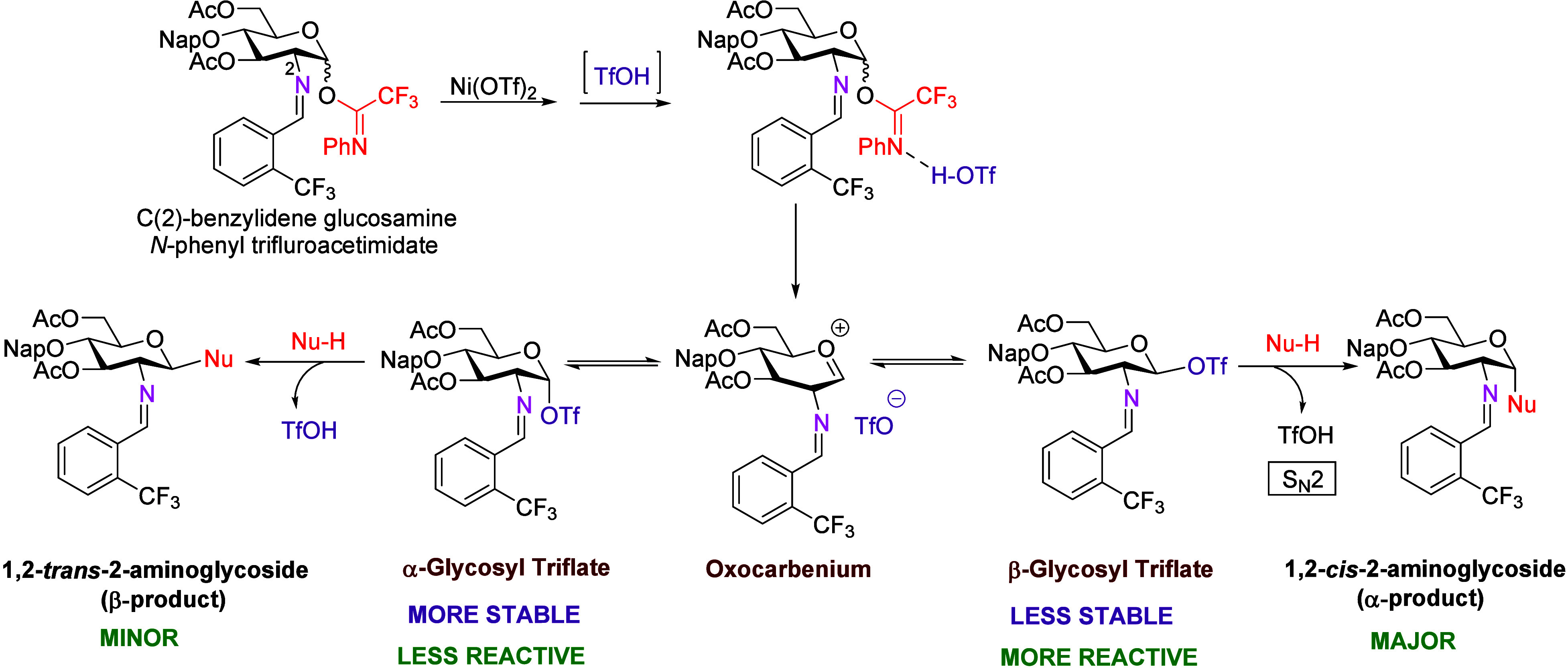
Proposed Mechanism for Triflic Acid-Catalyzed 1,2-*cis* Glycosylation

To access a single, selectively protected GlcNα­(1,4)­GlcA HS disaccharide that is compatible with site-selective 3-*O*- and 6-*O*-sulfation, *N*-sulfation, and *N*-acetylation, we designed a protection strategy that enables access to all relevant intermediates required for the corresponding glycopolymers. Accordingly, we strategically placed 2-naphthylmethyl (Nap) and acetyl (Ac) protecting groups on both the glucosamine-derived electrophilic donor **1** and the glucuronic acid nucleophilic acceptor **2** (Scheme 2). Based on our mechanistic studies,^3^ triflic acid was used in the glycosylation reaction, instead of Ni­(OTf)_2_, due to its operational simplicity. The use of 5 mol % triflic acid with C(2)-*N*-trifluoromethylbenzylidene glucosamine donor **1** provided excellent 1,2-*cis* selectivity and demonstrated excellent scalability, making it suitable for the large scale synthesis of the designed key disaccharide **3** (Scheme 2).^26^ Disaccharide **3** was diversified via *N*-benzylidene removal to afford **4** or selective 6-*O*-deacetylation to yield **5** (Scheme 2). Subsequent functionalization of **4** and **5** provided six disaccharide intermediates (**6**–**11**). Intermediate **4** underwent *N*-acetylation, *N*-trifluoroacetylation, 3-*O*-deacetylation and selective sulfation, followed by removal of the Nap group, to furnish **9**–**11** in overall good yields. The labile trifluoroacetyl group was hydrolyzed at late- stage to reveal the free amine. In parallel, disaccharide **5** was converted to **6** via *N*-benzylidene removal and simultaneous 6-*O*-/*N*-sulfation, or to **7** through 3-*O*-deacetylation and sulfation. For **8**, 6-*O*-Nap protection of **5** followed by sequential *N*-benzylidene removal, *N*-sulfation, 3-*O*-deacetylation, and sulfation, with final global Nap removal using DDQ, afforded disaccharide **8** (Scheme 2).^2^ The differentially sulfated disaccharides were individually subjected to copper-catalyzed azide–alkyne cycloaddition with ROMP-capable oxanorbornene polymerizable scaffolds **12**–**14**, followed by ester hydrolysis to afford monomers (M1–M4) specifically designed for polymerization (Figure 5).^1,2^

**Scheme 2:**
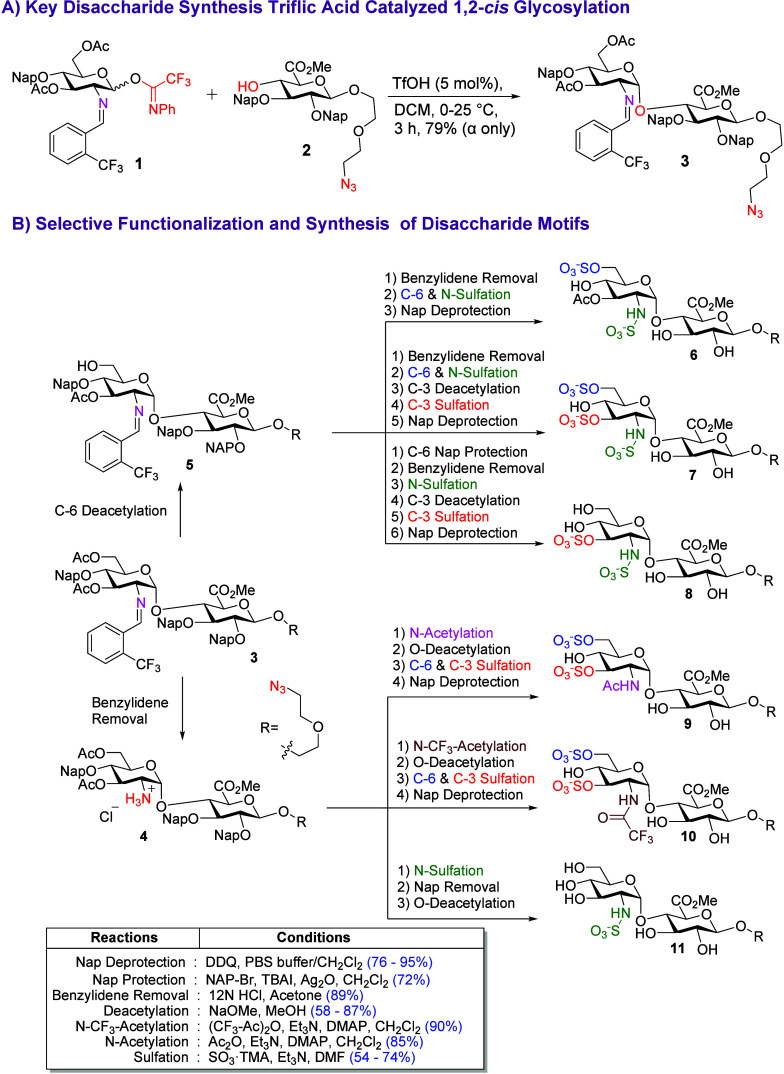
Stereoselective α-1,2-*cis*-Glycosidiation and Synthesis of Disaccharide Motifs for HS-Mimicking Glycopolymers

**Figure 6:**
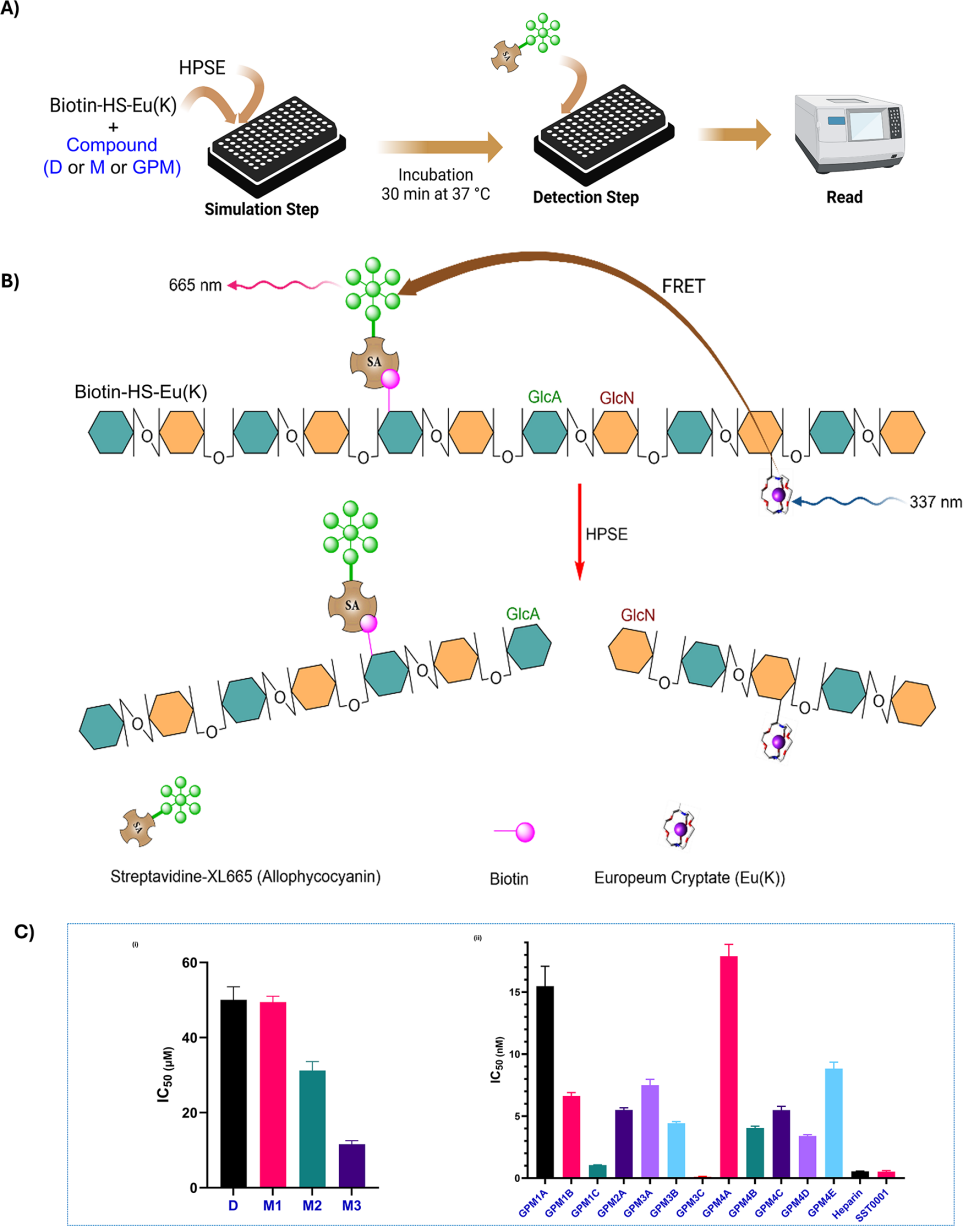
(A) TR-FRET assay detecting biotin–HS–cryptate cleavage by HPSE. (B) Inhibitors preventing cleavage, increasing FRET signal. (C) IC_50_ values (μM) of HS mimetics: disaccharide, D, and monomers, M. (D) IC_50_ values (nM) for GPM, heparin, and SST0001. ****p < 0.0001.

Ring-opening metathesis polymerization (ROMP) with the Grubbs third-generation catalyst (G3) was used to generate the neoglycopolymer (Figure 5). The degree of polymerization (DP_*n*_) and molecular weight of the polymer can be controlled by adjusting the catalyst loading. Traditional solvents like methylene chloride could not solvate the sulfated monomer, and aqueous systems required novel ruthenium catalysts. To overcome this challenge, we used a 2.5:1 cosolvent ratio of 2,2,2-trifluoroethanol and 1,2-dichloroethane, addressing solvent polarity issues and achieving complete polymerization in under 1 h with complete conversion. The monoantennary monomer M1, with catalytic loadings of 30, 10, and 7 mol %, gave GPM1A (*n* = 5), GPM1B (*n* = 9), and GPM1C (*n* = 18), respectively (Figure 5).^1^ The diantennary monomer M2, with 10 mol % G3, gave GPM2A (*n* = 8),^1^ while the diantennary monomer M3, with catalytic loadings of 20, 11.5, and 9 mol %, gave GPM3A (*n* = 5), GPM3B (*n* = 9), and GPM3C (*n* = 12), respectively.^25^ However, subjecting M3 to higher degrees of polymerization (*n* > 12) significantly decreased the yield of the resulting glycopolymer; as such, larger glycopolymers with *n* > 12 were not pursued. The diantennary monomers M4A to M4E, with varying sulfation patterns on GlcN and a catalytic loading of 9 mol % of G3, gave GPM4A (*n* = 12), GPM4B (*n* = 11), GPM4C (*n* = 10), GPM4D (*n* = 11), and GPM4E (*n* = 12), respectively (Figure 5).^2^

## 5 Heparanase Inhibition Activity

HPSE inhibition activity of synthesized glycopolymers and their monomers was assessed using a time-resolved fluorescence resonance energy transfer (TR-FRET) assay (Figure 6A). This assay employs FRET between a donor (europium cryptate) and an acceptor (streptavidin-XL665), resulting in an emission signal when the donor and acceptor are in proximity. Cleavage of the biotin-HS-cryptate substrate by HPSE decreases the FRET signal, whereas inhibitors prevent cleavage, maintaining a high signal and allowing quantitative evaluation of the enzyme activity (Figure 6B).

The GlcNS­(6S)­α­(1,4)­GlcA disaccharide D (Figure 5) was used as a reference and displayed a moderate IC_50_ value for HPSE inhibition (50.1 ± 3.46 μM), which was comparable to that of monomer M1 (49.4 ± 1.56 μM, Figure 6C). Remarkably, the neoglycopolymers derived from M1 (GPM1A, GPM1B, and GPM1C) exhibited progressively more potent inhibition in the nanomolar range (Figure 6D), with GPM1C showing the most potent activity (1.05 ± 0.02 nM), suggesting that increasing the polymer chain length enhances the inhibitory potency. The diantennary monomer M2 exhibited a better IC_50_ value (31.23 ± 2.36 μM) than M1 (Figure 6C), likely due to the additional GlcN residue, which enables stronger interaction with the HBD-2 region of HPSE (Figure 2B). This enhanced interaction was reflected in the neoglycopolymer GPM2A (*n* = 8), which showed a significant improvement in inhibition (5.49 ± 0.18 nM, Figure 6D).^1^ Interestingly, the third-generation diantennary monomer M3 (Figure 6C) displayed a markedly better IC_50_ value (11.59 ± 0.95 μM) than M1 and M2, likely due to the carboxylate group’s ability to interact effectively with HBD-2. This interaction was further reflected in the neoglycopolymers derived from M3, with GPM3A (*n* = 5) showing an IC_50_ of 7.49 ± 0.48 nM, GPM3B (*n* = 9) 4.43 ± 0.13 nM, and GPM3C (*n* = 12) achieving the lowest IC_50_ at picomolar range of 0.10 ± 0.036 nM, demonstrating the potential of the third-generation neoglycopolymers in HPSE inhibition (Figure 6D).^2,25^ Overall, the data indicate that monomer structure and polymer length are crucial in enhancing inhibitory potency, with GPM3C being the most potent candidate. We investigated the interaction of GPM3C (*n* = 12) with HPSE. As shown in Figure 7, a monomer unit is deeply embedded within the active site, while the remainder of the polymer surrounds the enzyme, particularly highlighting the HBD-2, which contains the most electropositive regions.^4^

**Figure 7:**
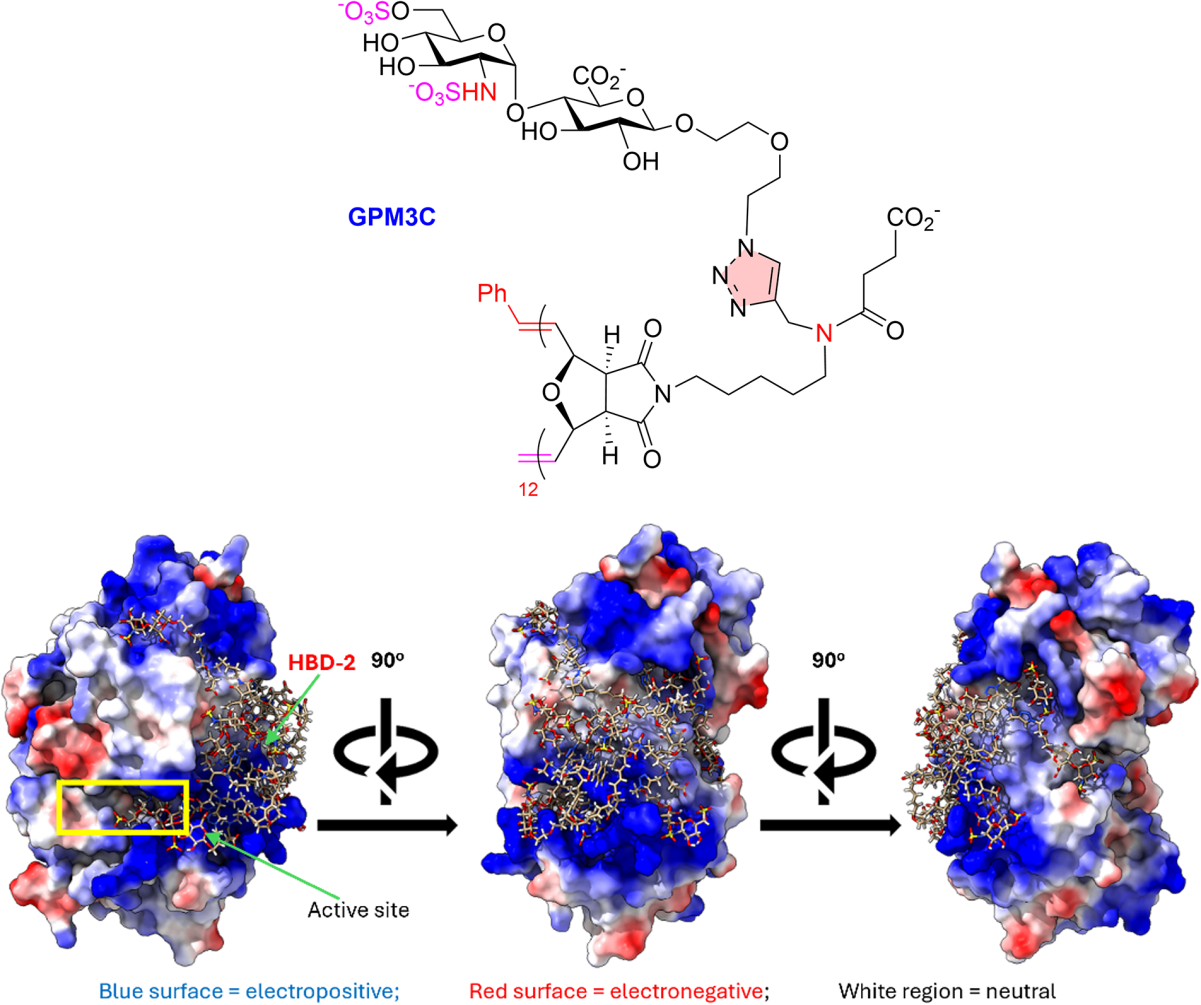
GPM3C (*n* = 12) docked into the HPSE crystal structure (PDB: 5E8M).^4^

**Table 1:** Binding Activity of Glycopolymer GPM3C with HS-Binding Proteins

entry	compd	HSBP	bioassay	response (nM)
1	GPM3C	PF4	BLI	1290 ± 0.44^a^
2	heparin	PF4	BLI	1.46 ± 0.09^a^
3	SST0001	PF4	BLI	5.04 ± 0.28^a^
4	GPM3C	FIIa	CSA	>4500^a^
5	heparin	FIIa	CSA	4.63 ± 0.22^a^
6	SST0001	FIIa	CSA	>900^a^
7	GPM3C	FXa	CSA	>4500^a^
8	heparin	FXa	CSA	266 ± 11.5^a^
9	SST0001	FXa	CSA	>900^a^
10	GPM3C	FGF-1	BLI	>2000^b^
11	heparin	FGF-1	BLI	4.6 ± 3.3^b^
12	GPM3C	FGF-2	BLI	691 ± 16.2^b^
13	heparin	FGF-2	BLI	0.15 ± 0.11^b^
14	GPM3C	VEGF	BLI	281 ± 16.2^b^
15	heparin	VEGF	BLI	4.91 ± 1.55^b^
16	GPM3C	P-selectin	BLI	351.5 ± 27.6^b^
17	heparin	P-selectin	BLI	124.8 ± 15.2^b^
18.	GPM3C	S1	BLI	13.1 ± 1.1^a^
19.	GPM3C	S-protein	BLI	32 ± 1.5^a^

a IC_50_ values.

b Apparent *K*_D_.

**Figure 8:**
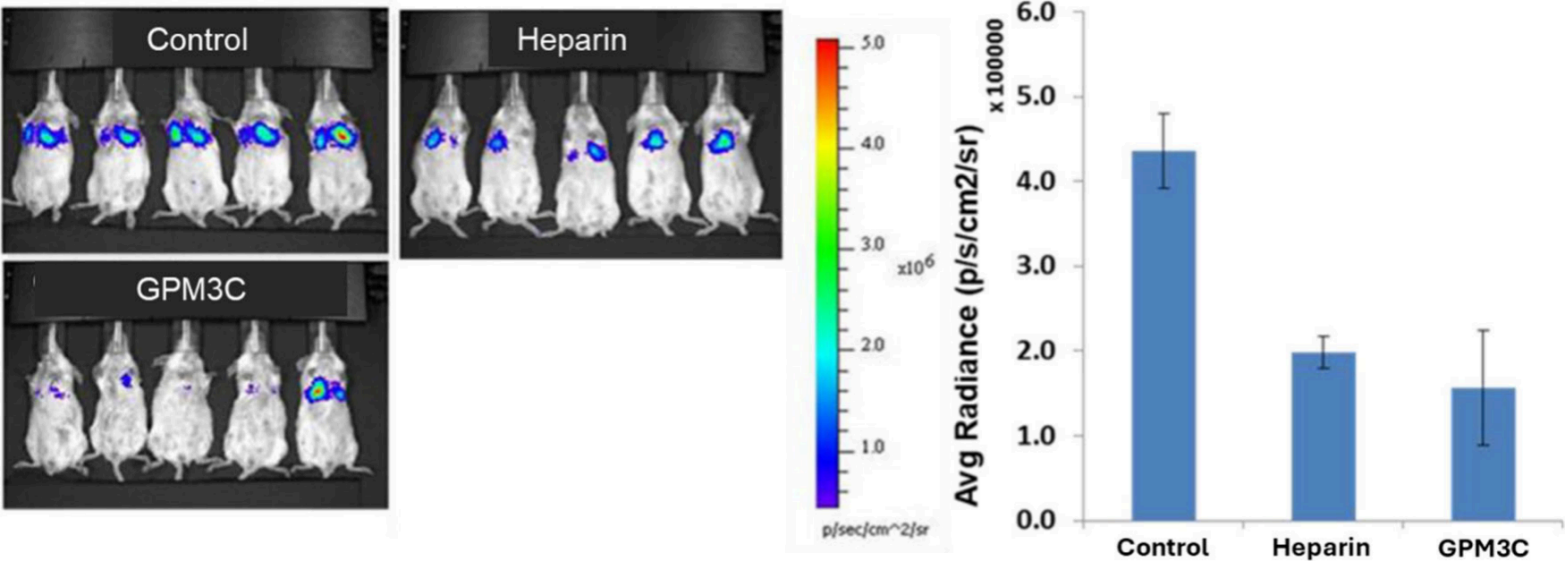
Effect of GPM3C on 4T1 metastasis. Luciferase-labeled 4T1 cells (1 × 10^5^ cells/mouse) were injected intravenously into mice (*n* = 6/group) with PBS, heparin, or GPM3C (100 μg/mouse). Bioluminescent imaging on day 7 measured tumor burden using photon counts and Living Image software. Adapted with permission from ref 2. Copyright 2019 American Chemical Society.

As GPM3C proved to be the most potent glycopolymer, we further investigated the effects of varying sulfation patterns on its GlcN residues, creating the diantennary monomers M4A-M4E (Figures 5 and 6D). We assessed the impact of removing of the 6-*O* sulfate group at the −2 subsite on HPSE inhibition by measuring the IC_50_ of GPM4A. The GlcNSα­(1,4)­GlcA disaccharide unit of GPM4A (*n* = 12) without the 6-*O* sulfate demonstrated the least inhibitory potency in this series with an IC_50_ of 17.9 ± 0.95 nM. Intriguingly, when 3-*O* sulfate group is added, GPM4B (*n* = 11) with GlcNS­(3S)­α­(1,4)­GlcA containing 3-*O* sulfate showed a better IC_50_ of 4.04 ± 0.15 nM. However, when both 3-*O* and 6-*O* sulfate groups are present, GPM4C (n = 10) with GlcNS­(3S, 6S)­α­(1,4)­GlcA having 3-*O*, 6-*O*, and 2-*N* sulfates showed a decrease in potency (5.48 ± 0.31 nM), suggesting that oversulfation is detrimental to the HPSE-HS interaction. To evaluate whether the 2-*N* sulfate group could be critically important for HPSE–HS interaction, we measured the IC_50_ of GPM4E (*n* = 12) with GlcN­(3S, 6S)­α­(1,4)­GlcA having both 3-*O* and 6-*O* sulfate and a free NH_2_ group on the GlcN unit. As predicted, its inhibitory potential is reduced with an IC_50_ of 8.83 ± 0.52 nM, suggesting the importance of 2-*N* sulfation for HPSE inhibition. Interestingly, when 3-*O* and 6-*O* sulfate groups are present, GPM4D (*n* = 11) with GlcNAc­(3S, 6S)­α­(1,4)­GlcA containing *N*-acetyl, showed an improved IC_50_ of 3.40 ± 0.10 nM. Overall, specific sulfation pattern is recognized to significantly influence interactions with HPSE, particularly the sulfation at the 6-*O* and C2-*N* positions of the GlcN sugar unit (Figure 6D). Consequently, GPM3C emerged as the best candidate among all the glycopolymers, demonstrating comparable potency relative to both heparin and SST0001 (Figure 6D).^2^

## 6 Affinity and Cross-Bioactivity with Other HS-Binding Proteins

The interaction of HS with HSBPs is a critical aspect of numerous biological and pathological processes. Therefore, it is imperative to evaluate the interactions and biological activities of HS mimetics with these proteins. Accordingly, we evaluated the cross-bioactivity of the most potent HPSE inhibitor GPM3C against several key HSBPs. We evaluated the potential inhibitory effects of GPM3C on key HSBPs: PF4, FIIa, FXa, FGF-1, FGF-2, VEGF, P-selectin, S1, and S-protein using two distinct bioassays (Table 1): biolayer interferometry (BLI) and chromogenic substrate assay (CSA).

PF4 is a small, cationic chemokine that is released by activated platelets. It plays a key role in blood coagulation, wound healing, and inflammation by binding to HS and heparin.^36^ Its strong affinity for heparin can also trigger immune responses, affecting platelet counts and increasing thrombotic risk.^37,38^ Another HSBP is the plasma protein ATIII, a natural anticoagulant protein. When heparin binds to ATIII, it induces a conformational change that enhances ATIII’s anticoagulant effects.^39^ Activated ATIII subsequently inactivates key clotting factors such as FXa and FIIa. Moreover, the binding of heparin and HS mimetics to angiogenic growth factors such as FGF-1, FGF-2, and VEGF released during ECM degradation by HPSE—has been shown to promote tumor growth.^40^ HS’s structural similarity to heparin poses challenges for using HS mimetics as HPSE inhibitors, as HPSE inhibitors’ interactions with HSBP make them unsuitable for targeted therapy.

GPM3C demonstrated an affinity for PF4 that is nearly 1000-fold lower than that of heparin (Table 1, entries 1 and 2). The reduced affinity can be attributed to the absence of IdoA-GlcN regions in GPM3C, which are essential for binding to PF4.^41–44^ Furthermore, GPM3C exhibits a lower affinity for PF4 when compared to SST0001, a known HPSE inhibitor (Table 1, entry 3). GPM3C also showed no significant anticoagulant activity (ATIII: Anti-FXa IC_50_ > 4500 nM; Anti-FIIa IC_50_ > 4500 nM).^25^ This result suggests that, like SST0001, GPM3C can potentially inhibit HPSE without the bleeding risks commonly associated with heparin-based therapies (Table 1, entries 4 and 7 vs entries 6 and 7). In contrast, heparin demonstrated a significantly higher affinity for both FXa and FIIa (Table 1, entries 5 and 8) than GPM3C and SST0001. Further, GPM3C exhibited low affinity for growth factors (FGF-1, FGF-2, and VEGF) (Table 1, entries 10, 12, and 14), suggesting that it may reduce the off-target effects often observed with other polymeric HS mimetics, which can inadvertently promote tumor growth.^2^ Additionally, GPM3C was observed to effectively bind to P-selectin (Table 1, entry 16),^2^ a protein responsible for P-selectin mediated tumor cell adhesion to endothelial cells, which can be suppressed by heparin (Table 1, entry 17).^45,46^ This result indicates that GPM3C can be a promising tool for targeted cancer therapy through HPSE and P-selectin inhibition.

The role of HS in mediating viral entry and egress, such as that of SARS-CoV-2, has been well established. The viral surface spike (S) glycoprotein of SARS-CoV-2 binds to the host cell’s angiotensin-converting enzyme-2 (ACE2) receptor to initiate virus entry.^47^ The S-protein contains multiple HBDs, including a prominent site at the S1/S2 junction, which overlaps with the furin cleavage site essential for viral activation.^36^ GPM3C demonstrated excellent binding affinity for the S1-protein (IC_50_ = 13 ± 1.1 nM) and S-protein (IC_50_ = 32 ± 1.5 nM) (Table 1, entries 18–19), which can be attributed to the preference of a C6-*O* and C2-*N* positions of the GlcN sugar unit.^48^ Furthermore, BLI assays revealed that compound GPM3C interacts with the S1/S2 junction (*K*_D_ = 13 ± 1.1 nM), confirming its ability to engage multiple HBDs on the spike protein.

## 7 Therapeutic Application: Antimetastatic Activity

The glycopolymer GPM3C shows promising inhibitory activity against HPSE. However, questions remain regarding how its effects will manifest both in vitro and in vivo, especially in relation to cancer metastasis. This complex process involves various factors, with one of the most critical being the degradation of the ECM, particularly the HS chains, which is driven by the HPSE enzyme. This degradation allows cancer cells to spread to distant organs, fueling tumor progression and contributing to treatment failure.

### 7.1 Breast Cancer Metastasis

Breast cancer is the leading cause of cancer-related mortality in women, accounting for a significant proportion of both new diagnoses and cancer deaths worldwide. In the pursuit of more effective treatments, one promising strategy is targeting HPSE to block metastasis. This led us to test the efficacy of GPM3C in a 4T1 mammary carcinoma model designed to replicate human breast cancer metastasis (Figure 8). GPM3C markedly inhibited the extravasation of 4T1 cells and their subsequent colonization of the mouse lungs, similar to the effect exerted by SST0001 (not shown).^2^ These results indicate that GPM3C inhibits the ability of blood-borne carcinoma cells to extravasate through the subendothelial basement membrane by modulating HPSE activity, which was further supported by a [^35^S] sulfate-labeled ECM degradation assay (not shown).^2^

### 7.2 Myeloma Tumor Growth and Metastasis

HPSE plays a crucial role in promoting the proliferation and survival of myeloma cells, making it a key therapeutic target.^49–51^ GPM3C decreased myeloma cell viability and downregulated HPSE mRNA expression and enzymatic activity in CAG cells (not shown).^4^ *In vivo* studies showed that HPSE inhibitor GPM3C significantly inhibited tumor growth in mice subcutaneously implanted with MPC-11 tumors, outperforming SST0001 and achieving a tumor growth inhibition (TGI) of 85.77%, compared to SST0001′s 64.78% (Figure 9a). GPM3C showed tumor growth inhibition comparable to Bortezomib, a standard treatment for multiple myeloma patients, through proteasome inhibition. Notably, GPM3C alone and its combination with Bortezomib enhanced tumor growth suppression (Figure 9b). In a more clinically relevant CAG human myeloma model, GPM3C reduced bone metastasis and inhibited myeloma cell growth in the bone marrow of NOD/SCID mice (Figure 9c), reinforcing its potential as a therapeutic agent for targeting myeloma through HPSE inhibition. These findings position GPM3C as a promising therapeutic candidate for multiple myeloma, with the potential to synergize with treatments like Bortezomib, particularly for patients who develop drug resistance.^4^

**Figure 9:**
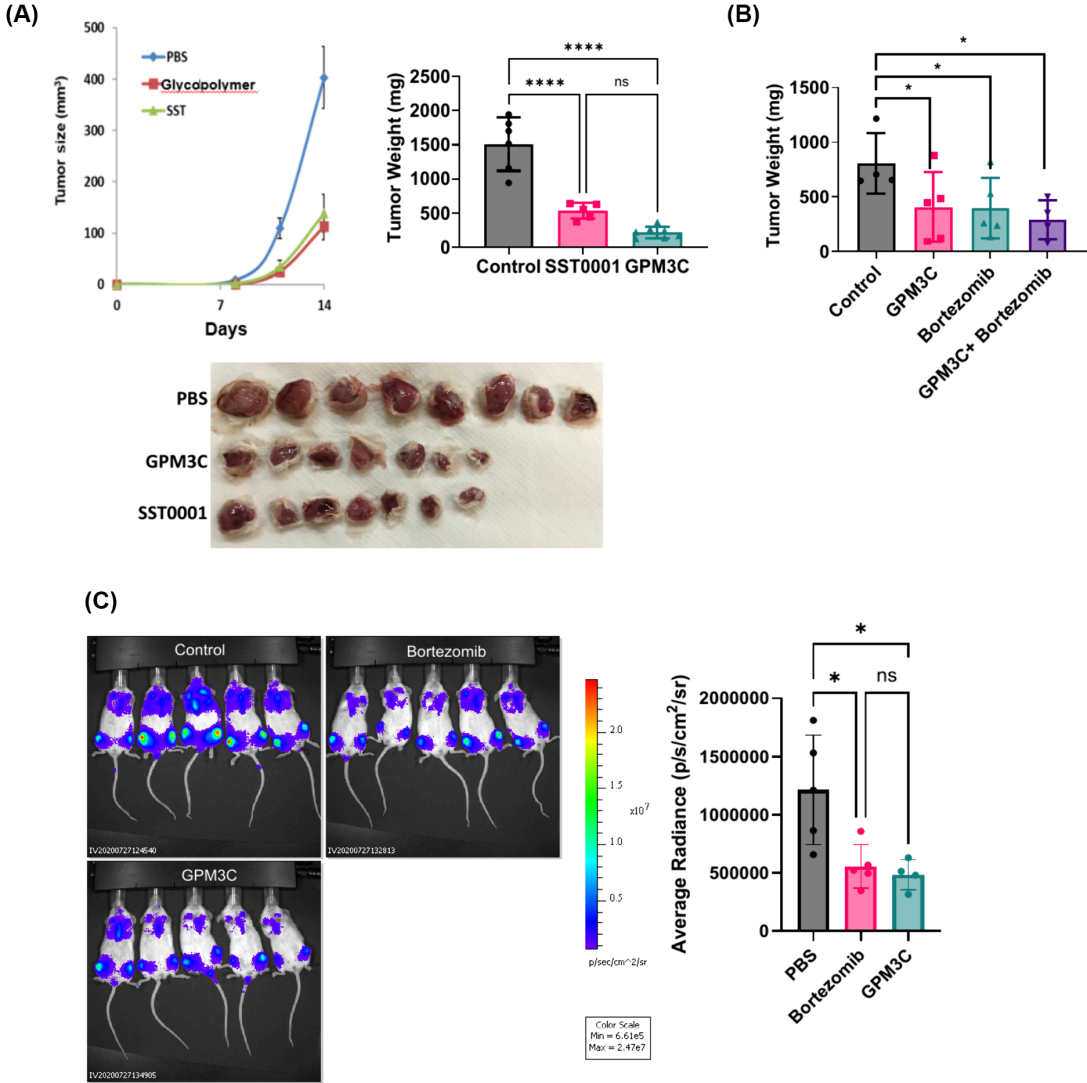
(A) GPM3C inhibiting MPC-11 tumor growth more effectively than SST0001. (B) GPM3C showing comparable efficacy to Bortezomib. (C; left) IVIS images of luciferase-expressing CAG myeloma cells in bones of treated mice. (C; right) GPM3C reducing bone tumor burden, with efficacy comparable to bortezomib. ****p < 0.0001; *p = 0.05. Adapted with permission from ref 4. Copyright 2025 American Chemical Society.

## 8 Therapeutic Application: Β-Cells Protection in Diabetes

Diabetes is a chronic condition characterized by high blood glucose levels due to ineffective insulin production or insulin resistance, with the loss of insulin-producing β-cells.^52^ Islet HS is crucial for β-cell survival, and its cleavage by HPSE contributes to β-cell death, inflammation, and oxidative stress.^53^ GPM3C has demonstrated potent protective effects on pancreatic β-cells and human islets against HPSE-induced damage. In vitro studies showed that GPM3C (0.3 μM) effectively preserved cell viability, morphology, and islet-like colony formation of the mouse Min-6 β-cell line, counteracting the detrimental effects of HPSE (5 ng/mL) (Figure 10A). Ex vivo treatment of human pancreatic islets with GPM3C (0.3 μM) restored HS content, which is typically degraded by HPSE, as shown by Alcian blue staining and FITC-conjugated anti-HS antibody fluorescence imaging (Figure 10B,C). Additionally, GPM3C reduced the expression of pro-inflammatory cytokines, including TNFα, IL1β, and IL8, suggesting its ability to suppress inflammation in human pancreatic islets (not shown). These findings underscore GPM3C’s potential as a therapeutic agent to protect β-cells and islets from HPSE-mediated HS degradation and inflammation, offering a promising strategy for treating diabetes.^26^

**Figure 10:**
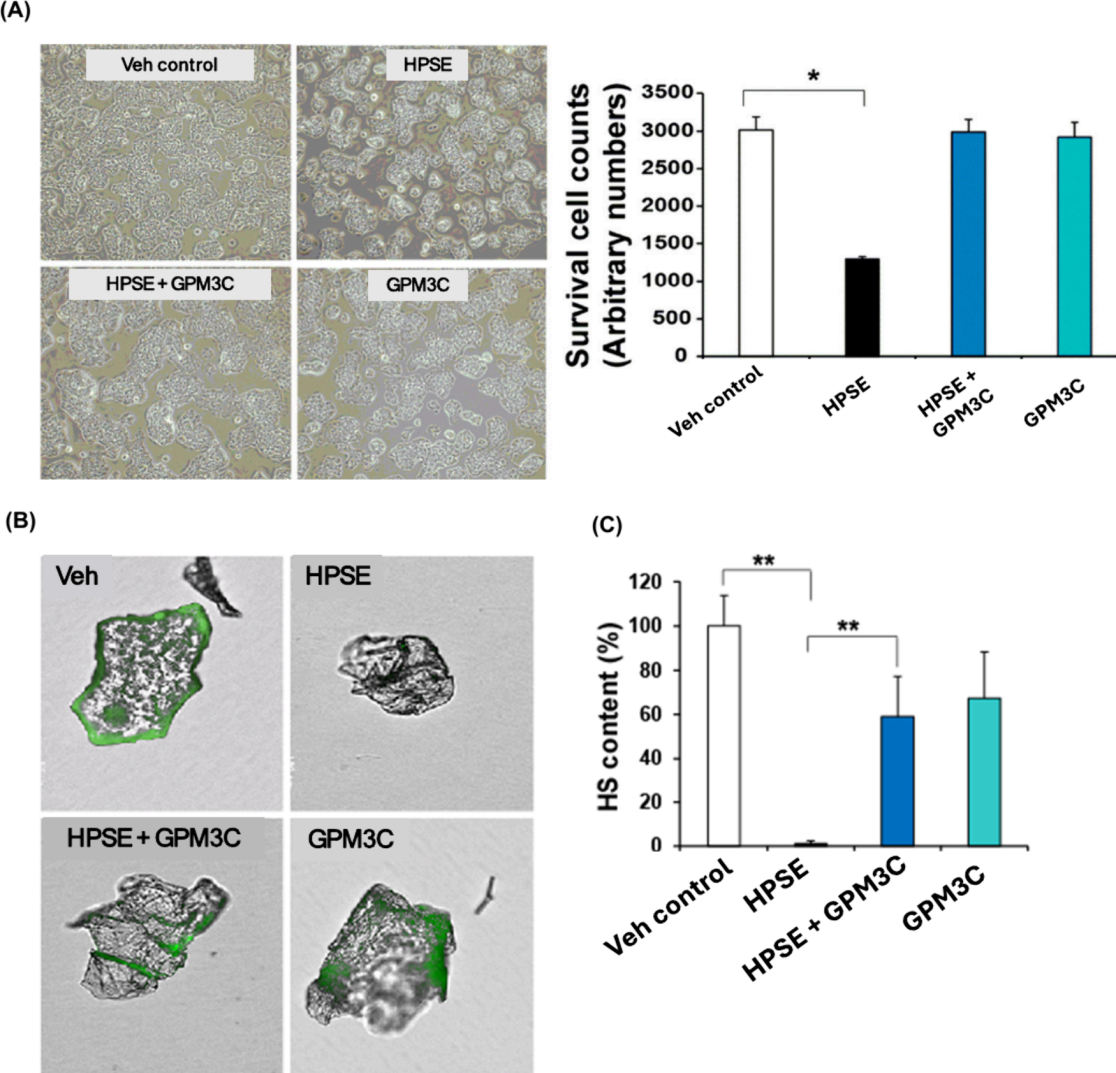
(A) Mouse Min-6 β-cells treated with PBS, HPSE, HPSE + GPM3C, or GPM3C for 24 h and assessed by Trypan-Blue. *p < 0.05. (B) Human pancreatic islets treated with vehicle, heparanase (10 ng/mL), heparanase + GPM3C, or GPM3C (0.3 μM) for 24 h. Islets were stained with FITC-conjugated anti-HS antibody. (C) HS fluorescence intensity quantified using ImageJ. Data are mean ± SEM (*n* = 5 or 6), **p < 0.01. Adapted with permission from ref 26. Copyright 2022 American Chemical Society.

## 9 Summary

This comprehensive work presents an innovative approach to the design and synthesis of glycopolymers as HS mimetics that target heparanase, an enzyme crucial for modulating HS-associated physiological conditions with high affinity and specificity. The disaccharide cores of these glycopolymers were synthesized employ our method of the catalytic stereoselective 1,2-*cis*-2-amino glycosylation strategy. These glycopolymers were designed with control of their degree of polymerization and were functionalized with glycan residues bearing well-defined sulfation patterns, which influence their selectivity for HPSE over other HSBPs. The degree of polymerization and sulfation pattern, specifically the sulfation at 2-*N* and 6-*O* positions, significantly affect the affinity and activity of the glycopolymers against HPSE. The most potent glycopolymer, GPM3C, showed remarkable *in vivo* activity for mitigating metastasis and tumor growth in mammary carcinoma and multiple myeloma through inhibiting HPSE. GPM3C also protected pancreatic β-cells and preserved HS contents in human islets from HPSE-induced damage and degradation. Additionally, GPM3C demonstrated promise as an inhibitor of HSPG-mediated endocytosis of SARS-CoV-2, blocking viral attachment to host cells by binding to the viral spike protein. Importantly, GPM3C exhibited minimal affinity for off-target HSBPs, significantly reducing the risk of adverse effects. These results establish synthetic glycopolymers as potent and selective HS mimetics and provide valuable insights for the design of the next generation of polymeric HS mimetics, enabling the precise targeting of specific HSBP through the strategic control of their size, multivalency, and sulfation patterns.

## Data Availability

Data discussed in Figure 4 found in refs 16 and 17; Scheme 1, ref 19; Figure 5, refs 16, 17, and^18^ ; Figure 6, refs 16, 17, and^18^ ; Figure 7, ref 21; Table 1, refs 16 and 17 ; Figure 8, ref 16; Figure 9, ref ; Figure 10, ref 19; and Figure 11, ref 32.
